# Immune response dynamics and *Lutzomyia longipalpis* exposure characterize a biosignature of visceral leishmaniasis susceptibility in a canine cohort

**DOI:** 10.1371/journal.pntd.0009137

**Published:** 2021-02-22

**Authors:** Manuela da Silva Solcà, Maiara Reis Arruda, Bruna Martins Macedo Leite, Tiago Feitosa Mota, Miriam Flores Rebouças, Matheus Silva de Jesus, Leila Denise Alves Ferreira Amorim, Valéria Matos Borges, Jesus Valenzuela, Shaden Kamhawi, Patrícia Sampaio Tavares Veras, Deborah Bittencourt Mothé Fraga, Claudia Ida Brodskyn

**Affiliations:** 1 Instituto Gonçalo Moniz—Fundação Oswaldo Cruz, Bahia, Brazil; 2 Escola de Medicina Veterinária e Zootecnia—Universidade Federal da Bahia, Bahia, Brazil; 3 Instituto de Matemática e Estatística—Universidade Federal da Bahia, Bahia, Brazil; 4 Vector Molecular Biology Section, Laboratory of Malaria and Vector Research, National Institute of Allergy and Infectious Diseases, National Institutes of Health, Rockville, Maryland, United States of America; 5 Instituto Nacional de Ciência e Tecnologia de Doenças Tropicais / INCT-DT, Bahia, Brazil; 6 Instituto Nacional de Ciência e Tecnologia-Investigação em Imunologia /INCT-III, São Paulo, Brazil; University of Iowa, UNITED STATES

## Abstract

**Background:**

Reports have shown correlations between the immune response to vector saliva and Leishmaniasis outcome. We followed dogs in an endemic area for two years characterizing resistance or susceptibility to canine visceral leishmaniasis (CVL) according to *Leishmania infantum* diagnosis and clinical development criteria. Then, we aimed to identify a biosignature based on parasite load, serum biological mediators’ interactions, and vector exposure intensity associated with CVL resistance and susceptibility.

**Methodology/Principal findings:**

A prospective two-year study was conducted in an area endemic for CVL. Dogs were evaluated at 6-month intervals to determine infection, clinical manifestations, immune profile, and sandfly exposure. CVL resistance or susceptibility was determined upon the conclusion of the study. After two years, 78% of the dogs were infected with *L*. *infantum* (53% susceptible and 47% resistant to CVL). Susceptible dogs presented higher splenic parasite load as well as persistence of the parasite during the follow-up, compared to resistant ones. Susceptible dogs also displayed a higher number of correlations among the investigated biological mediators, before and after infection diagnosis. At baseline, anti-saliva antibodies, indicative of exposure to the vector, were detected in 62% of the dogs, reaching 100% in one year. Higher sandfly exposure increased the risk of susceptibility to CVL by 1.6 times (CI: 1.11–2.41). We identified a discriminatory biosignature between the resistant and susceptible dogs assessing splenic parasite load, interaction of biological mediators, PGE_2_ serum levels and intensity of exposure to sandfly. All these parameters were elevated in susceptible dogs compared to resistant animals.

**Conclusions/Significance:**

The biosignature identified in our study reinforces the idea that CVL is a complex multifactorial disease that is affected by a set of factors which are correlated and, for a better understanding of CVL, should not be evaluated in an isolated way.

## Author summary

Visceral Leishmaniasis (VL) is a disease that can affect humans and dogs, caused by a parasite called *Leishmania* transmitted through the bite of sandfly insects. During the bite, together with the parasite, the insects also inoculate their saliva into the host. The host immune response produces molecules to the sandfly saliva, such as antibodies and cytokines that can impact VL resistance or susceptibility. The presence of these molecules also indicates if the insects bit the hosts. We followed dogs of a VL endemic area for two years to study Canine Visceral Leishmaniasis (CVL) and immune response to sandfly saliva. Dogs were evaluated at 6-month intervals to determine *Leishmania* infection, clinical manifestations, parasite load, immune response, and sandfly exposure. CVL resistance or susceptibility was determined upon the conclusion of the study. Dogs living in the endemic area were intensely bitten, as at the beginning of the study, 62% of the dogs present anti-saliva antibodies, reaching 100% after one year. Our findings revealed a biosignature of CVL susceptibility characterized by elevated parasite load, interaction of cytokines, and higher exposure to the sandfly. This data reinforced that CVL is a complex disease affected by several factors related to each other.

## Introduction

Visceral leishmaniasis (VL), a zoonosis caused by the protozoan *Leishmania infantum*, is transmitted through the bite of *Lutzomyia longipalpis* sandflies [1–3]. On feeding, infected vectors inoculate metacyclic promastigote forms of *Leishmania* along with their saliva [4]. The immunomodulatory activity and anticoagulant, antiplatelet and anti-inflammatory properties of sandfly salivary proteins favor the establishment of parasite infection [5–8].

Mice immunized with vector salivary proteins demonstrated protection against *Leishmania* infection [9,10]. However, other evidence showed that pre-exposure to *Lutzomyia intermedia* saliva increased *Leishmania braziliensis* infection severity [11]. More recent studies indicate that anti-saliva antibodies may be associated with increased VL severity in naturally infected dogs [12], and anti-saliva antibodies correlated with transmission intensity in dogs naturally infected by *L*. *infantum* [13,14].

Dogs infected with *L*. *infantum* show a wide range of clinical manifestations, varying from absence to severe disease presentation [15]. Clinical outcome is directly associated with resistance and susceptibility to canine VL (CVL) [16]. These features are highly correlated with the predominance of Th1 (IFNγ, IL-2, and TNFα) or Th2 (IL-4, IL-5, IL-10, IL-13, and TGFβ) immune responses, respectively [17,18]. A cross-sectional study identified distinct biomarkers in dogs. Animals displaying severe disease were associated with lower serum concentrations of LTB_4_ and PGE_2_ and higher levels of CXCL1 and CCL2. The combination of these mediators distinguished dogs with different clinical scores [12].

The present cohort study followed dogs in an endemic area for two years, characterizing resistance or susceptibility to CVL according to *L*. *infantum* infection diagnosis and clinical development criteria. Then, we aimed to identify a biosignature based on parasite load, serum biological mediators’ interactions, and vector exposure intensity associated with CVL resistance and susceptibility.

## Methods

### Ethics statement

The present study was approved by the local Institutional Review Board for Animal Experimentation (IGM-FIOCRUZ, protocol no. 007/2013) and was conducted in compliance with Brazilian Federal Law on Animal Experimentation (Law no. 11794). Informed written consent was obtained from all dog owners.

### Cohort design

A prospective 24-month cohort study was conducted from February 2014 to November 2017 in the municipality of Camaçari (latitude: 12°41′51′′S; longitude: 38°19′27′′W), endemic for both human VL and CVL, located in the state of Bahia, Brazil. Dogs were evaluated at baseline and then reevaluated at six-month intervals to determine *L*. *infantum* infection’s establishment, clinical manifestations of CVL, and vector exposure. In order to be included, each dog had to be reexamined at no less than two follow-up points after baseline evaluation.

### Diagnosis of *L*. *infantum* infection

To perform *L*. *infantum* infection diagnosis, splenic aspirate, skin biopsy, and whole blood samples were collected at regular 6-month intervals. Ten mL of blood were collected by cephalic puncture. Both splenic and skin samples were collected after performing trichotomy under cutaneous anesthesia with 500μL of 1% lidocaine hydrochloride (Hypofarma, São Geraldo, MG—Brazil). An ultrasound-guided biopsy was employed for the collection of splenic aspirates to avoid major splenic vessels. Two mL of splenic aspirates were collected by puncturing the spleen’s central region using a 25-gauge needle linked to a 20mL syringe. Skin biopsies were collected from healthy skin, without any lesions, at the lateral scapular region using a sterile 3 mm punch (Kolplast, Brazil) in all dogs. Parasitological evaluations were performed in splenic aspirates [19], and molecular evaluations using qPCR were performed employing DNA extracted from splenic aspirates, skin biopsies, and whole blood to assess tissue positivity and parasite load [20]. Anti-*Leishmania* antibodies were serologically detected using a chromatographic immunoassay (DPP LVC, Bio-Manguinhos, Rio de Janeiro, Brazil), followed by an ELISA confirmation assay (EIE LVC, Bio-Manguinhos, Rio de Janeiro, Brazil), according to Brazilian Ministry of Health standards [21]. Dogs were considered infected when they were positive, in at least one of the study time points, by serological, parasitological, or molecular assays in at least one of the tissues evaluated. When a given dog was diagnosed as infected, that animal was then considered infected at all subsequent time points. Animals were classified as negative when diagnostic test results were negative for *L*. *infantum* infection at all time points evaluated, regardless of clinical scores.

### Hemoparasite antibodies evaluation

To detect babesiosis and ehrlichiosis, specific serology by ELISA was performed on serum samples randomly selected from 41% (77/189) of the evaluated dogs. The serological analyses were performed in the Laboratory of Immunoparasitology at UNESP (Jaboticabal, SP, Brazil) according to Braga *et al*. and Gottlieb *et al* [22,23]. Detection of IgG antibodies indicated infection by *Ehrlichia canis* or *Babesia canis*.

### Serum albumin and globulin protein assessment

Serum samples randomly selected from 62% (92/148) of *L*. *infantum* infected dogs were employed to evaluate the concentration of globulin, albumin, and albumin-globulin ratio during the follow-up (every 6 months) using a colorimetric method with an A15 auto-analyzer (BioSystems, Barcelona, Spain).

### Clinical follow-up

Table 1 displays the clinical parameters related to canine visceral leishmaniasis that were assessed at each time point. Each parameter assessed was assigned a score in accordance with presence and intensity. A total clinical score was then calculated for each dog using the sum of all individual parameters. This composite score could range from 0 to 24 points [12].

**Table 1:** Canine visceral leishmaniasis clinical parameters employed to calculate the clinical score of each animal included in the study.

Clinical signs	Score based on intensity		
	0	1	2
Nutritional status	Normal or obese	Emaciate	Cachectic
Mucosa color	Normal	Pale	---
Periocular dermatitis	Absent	Around one eye	Present around two eyes
Crust on ears	Absent	Present in one ear	Present in two ears
Ear Ulcers	Absent	Present in one ear	Present in two ears
Muzzle Depigmentation	Absent	In less than 1/3 of the muzzle	In more than 1/3 of the muzzle
Muzzle Hyperkeratosis	Absent	In less than 1/3 of the muzzle	In more than 1/3 of the muzzle
Muzzle Lesions	Absent	Initial mucous lesion	Larger ulcerated lesion
Spleen size	Not palpable	Enlarged	---
Onychogryphosis	Absent	Slight enlargement	Excessive enlargement
Alopecia	Absent	Focal	In more than 1/3 of the body
Seborrheic dermatitis	Absent	Focal	In more than 1/3 of the body
Lymphadenomegaly	Absent	One or two enlarged lymph nodes of the same pair	Enlarged lymph nodes of different pairs

### Classification of resistance and susceptibility to CVL

At the end of the 24-month follow-up, animals infected by *L*. *infantum* were retrospectively classified as resistant or susceptible to CVL according to the evolution of clinical manifestations during the study period. In order to be classified as resistant or susceptible, dogs had to be examined at no less than two follow-up points after receiving an initial diagnosis of *L*. *infantum* infection. Classification of resistance was designated if 1) dogs received a diagnosis of *L*. *infantum* infection with a clinical score ≤ 3 and remained so or presented improvements at all subsequent follow-up evaluations; 2) *L*. *infantum*-infected dogs with clinical scores > 3 later exhibited a reduction ≤ 3 at two or more consecutive follow-up evaluations. Dogs were classified as susceptible when presenting clinical scores > 3 at two or more follow-up evaluations after *L*. *infantum* infection was first diagnosed.

### Biological mediator characterization

For the evaluation of biological mediators, we selected dogs in which *L*. *infantum* infection was first diagnosed at least 6 months after they were enrolled in the study, and they were also followed for more than 6 months after the first infection diagnosis. To be considered in the biological mediator characterization, a given dog had to remain negative for at least six months (two-time points) prior to being diagnosed with *L*. *infantum* infection and then be reevaluated twice after testing positive. The following biological mediators were measured in the sera of infected dogs by the Luminex assay (Milliplex Map Kit, USA): IFNγ, IL-10, TNFα, IL-2, IL-6, IL-7, IL-15, IL-8, CCL2, CXCL10, GM-CSF, and CXCL1. Leukotriene (LTB_4_) and Prostaglandin E_2_ (PGE_2_) concentrations were measured by ELISA (Cayman Chemical, USA) as described elsewhere [12,24]. Serum levels of biological mediators were compared between time points and also between resistant and susceptible groups. Additionally, we examined the relationships between the biological mediators within resistant and susceptible dogs before and after infection diagnosis using network analysis based on statistically significant Spearman correlations (*p* < 0.05).

### Evaluation of sandfly exposure

Exposure to sandflies was assessed by evaluating anti-saliva antibody production using the rLJM11 + rLJM17 (rLJM11+17) salivary proteins as antigens, following a previously described protocol [12,25]. rLJM11+17 proteins were first validated using an indirect ELISA to detect antibodies against *Lu*. *longipalpis* salivary gland sonicate (SGS) as a gold standard. SGS indirect ELISA protocol was performed as described elsewhere [25]. We used 177 dog sera, randomly selected from the endemic area, to validate rLJM11+17 salivary proteins.

Reactivity index (RI) values were calculated as the ratio between ELISA optical density readings of a given sample divided by the cut-off value for controls in its respective microplate [26]. Dogs were considered exposed to sandfly saliva when presenting an RI > 1. Any dogs initially determined as exposed to sandfly saliva were considered as exposed at all subsequent time points.

Sandfly exposure intensity scores were calculated by dividing the RI obtained at each follow-up by the lowest RI value recorded at any previous time points. Dogs displaying an exposure intensity ratio ≥ 2 were considered as highly exposed animals (presenting higher anti-saliva antibody levels), whereas animals with a ratio < 2 were considered as having a lower exposure to sandflies (presenting lower anti-saliva antibody levels).

### Statistical analysis

The assumption of normality of all quantitative variables was assessed by the Shapiro-Wilk test. Fisher’s exact or Pearson Chi-square test (*X*^2^) were used to compare canine cohort demographic characteristics between dogs that completed 24-month follow-up and those that did not and compare hemoparasite infection amongst negative and CVL-resistant and susceptible dogs. The concentration of globulin, albumin, and albumin-globulin ratio were described using median and interquartile range (IQR) and compared between resistant and susceptible groups at 6, 12, 18, and 24 months after infection diagnosis, employing Mann-Whitney test.

Frequencies of positive qPCR results amongst tissues and between groups were compared using Fisher’s exact or Pearson *X*^2^ tests. We analyzed CVL-resistant and susceptible dogs considering parasite load and the persistence of positive results employing qPCR. Dogs followed at least two times points after the first positive qPCR detection were suitable for persistence analysis. Persistent dogs were animals presenting qPCR positive results in most of the time points evaluated sequentially. Mann-Whitney test was employed to compare parasite loads between resistant and susceptible dogs at the time of infection diagnosis by qPCR, 6, 12, 18, and 24 months after infection diagnosis for each tissue evaluated.

Robust rank-based methods for analyzing longitudinal data were used to compare serum levels of biological mediators between CVL-resistant and susceptible groups across time [27]. Biological mediators were measured in the serum of each dog at 3 time points: 1) Before Infection Diagnosis (BID): 6 to 12 months prior to the first detection of *L*. *infantum* infection; 2) Infection Diagnosis (ID): when infection was first diagnosed; 3) After Infection Diagnosis (AID): 6 to 12 months after diagnosis of the infection. We used nonparametric procedures for conducting two-way ANOVA for repeated measures and tested the hypothesis of an interaction between time and group [28]. The relative treatment effects (RTE) were estimated and displayed with corresponding 95% confidence intervals. An increase in these effects over time indicates the increase in the levels of the biological mediators. The analyses were implemented using package nparLD in software R [27].

Receiver-Operator Characteristic (ROC) curve analysis was used to test the power of PGE_2_ to distinguish CVL-resistant and susceptible dogs, the Area Under the Curve (AUC) was determined for the best cut-off value.

Correlations between biological mediators were assessed using Spearman’s rank correlation coefficient before and after infection diagnosis in CVL-resistant and susceptible dogs. A heatmap was generated to represent the number of strong correlations presenting r ≥ 0.6 or r ≤ -0.6; *p* < 0.05 for each parameter. Cytoscape v3.7.2 software [29] was used to perform network analysis (host interactome) of statistically significant strong correlations between the evaluated mediators. Network densities of the biological mediators were also compared between resistant and susceptible dogs, before and after infection diagnosis.

Using SGS ELISA results as the gold standard, accuracy parameters with a 95% confidence interval (CI) were calculated for validation of rLJM11+17 ELISA. Spearman’s rank correlation coefficient, Kappa coefficient, and Pearson *X*^2^ between the two techniques were also calculated. Kruskal-Wallis and Dunn’s multiple comparison tests were used to compare RI values from rLJM11+17 ELISA between dogs classified with higher and lower sandfly exposure at each follow-up time point. Pearson *X*^2^ trend analysis was used to evaluate the distribution of frequencies between higher and lower sandfly exposure and CVL resistance and susceptibility. Relative risk (RR) at a 95% CI was calculated to determine susceptibility risk for CVL. Statistical analyses were performed using JMP statistical software v13 (SAS, Cary, NC, USA) and GraphPad Prism v5 (GraphPad Prism Inc., San Diego, CA). Results were considered statistically significant when *p* < 0.05.

## Results

### Canine cohort dynamics

Of a total of 285 dogs initially recruited at baseline, 66% (189) of the dogs met the criteria for inclusion in the analyses. Table 2 describes the demographic characteristics of the analyzed canine population. Dogs included were of both genders, 87.3% were mixed breed, the majority (80.5%) were of medium and large sizes, 71.4% were considered young or young adults, and 70.9% were domiciled.

**Table 2:** Canine characteristics recorded at study baseline, and animal groups classified at the end of follow-up, discriminating dogs evaluated until the end of the cohort (24 months).

Characteristics	Dogs followed until the end of the cohort (24 months)				Total (n = 189)		*p*-value*
	No (n = 86)		Yes (n = 103)				
	N	%	N	%	N	%	
**Sex**							0.2819
Female	40	46.5%	56	54.4%	96	50.8%	
Male	46	53.5%	47	45.6%	93	49.2%	
**Breed**							0.1767
Mixed breed	72	83.7%	93	90.3%	165	87.3%	
Pure breed	14	16.3%	10	9.7%	24	12.7%	
**Size**							0.6033
Toy (<2kg)	1	1.2%	0	0.0%	1	0.5%	
Small (2 to <10kg)	14	16.3%	11	10.7%	25	13.2%	
Medium (10 to <20kg)	37	43.0%	51	49.5%	88	46.6%	
Large (20 to <40kg)	29	33.7%	35	34.0%	64	33.9%	
Extra-large (>40kg)	5	5.8%	6	5.8%	11	5.8%	
**Age**							0.4453
Puppy (<1y)	11	12.8%	15	14.6%	26	13.8%	
Young (1 to < 2y)	31	36.0%	46	44.7%	77	40.7%	
Young adult (2 to < 3y)	28	32.6%	30	29.1%	58	30.7%	
Adult (3 to < 6y)	16	18.6%	12	11.7%	28	14.8%	
**Dog’s role in the household**							0.7093
Exclusively companion	22	25.6%	27	26.2%	49	25.9%	
Exclusively guardian	10	11.6%	16	15.5%	26	13.8%	
Both companion and guardian	54	62.8%	60	58.3%	114	60.3%	
**Lifestyle**							0.2793
Domiciled	57	66.2%	77	74.8%	134	70.9%	
Semi-domiciled	28	32.6%	26	25.2%	54	28.6%	
Stray	1	1.2%	0	0.0%	1	0.5%	
**Animal groups**							0.0768
Negative	25	29.1%	16	15.5%	41	21.7%	
Resistant	28	32.6%	42	40.8%	70	37%	
Susceptible	33	38.3%	45	43.7%	78	41.3%	

* Fisher’s exact or Pearson *X*^2^

**Fig 1:**
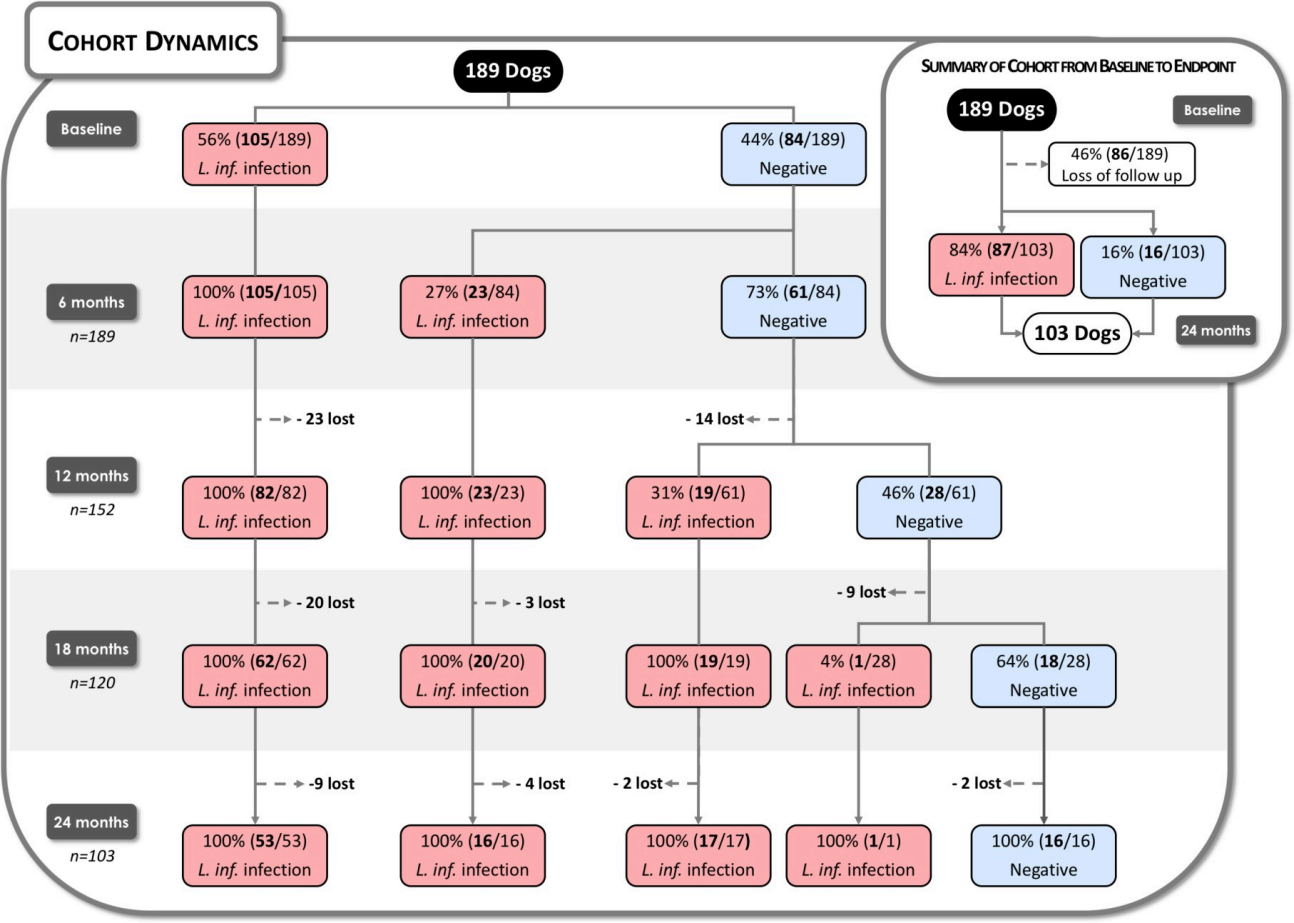
Cohort Dynamics. A total of 189 dogs were followed for 24 months, with *Leishmania infantum* infection assessed at six-month intervals. Red rectangles represent dogs presenting *L*. *infantum* infection at each follow-up point, while blue rectangles depict uninfected evaluated animals. Dotted lines represent a loss to follow-up. Summary of the cohort from baseline to endpoint reveals a 46% loss during the study, i.e., 54% remained throughout the two-year follow-up period.

Of 189 dogs, 56% (105/189) were infected with *L*. *infantum* at baseline, while 44% (84/189) tested negative. Six months later, 27% (23/84) of the negative dogs presented *L*. *infantum* infection, and after 12 months, 31% (19/61) of the negative dogs became infected (Fig 1). In summary, 78% (148/189) of the dogs were infected, and 41 animals were not. However, at the end of the 24-month follow-up, only 8% (16/189) of the dogs remained uninfected, while the remaining 25 dogs were lost to follow-up.

Among infected dogs, 47% (70/148) were considered resistant to CVL, while 53% (78/148) were considered susceptible. S1 Fig displays the clinical score profile during follow-up of resistant and susceptible dogs. During the study period, 46% (86/189) of dogs were lost to follow-up (Fig 1). A total of 103 dogs were followed until the end of the study period, and 84% (87/103) of these were considered *L*. *infantum*-infected; 48% (42/87) were resistant to CVL, while 52% (45/87) were susceptible. (Fig 1). No significant differences were found among dogs that completed the follow-up and those that did not complete, considering canine characteristics or group classification. (Table 2).

Concerning the hemoparasite infection, we found high proportions of positivity in all groups evaluated: 89% (8/9) in the negative group, 89% (31/35) in CVL-resistant dogs, and 97% (32/33) in susceptible ones. There was no statistically significant difference between the groups analyzed (*p* = 0.4; Pearson *X*^2^).

Regarding serum albumin and globulin assessment, evaluated CVL-susceptible dogs presented significantly lower serum albumin-globulin ratios compared to resistant animals during the follow-up (S1 Table).

### Molecular identification of *L*. *infantum* and parasite load quantification

In most of the dogs was confirmed CVL (93.9%), and it was possible to measure *L*. *infantum* DNA at least in one tissue at one or more of the time points during follow-up. Higher frequencies of parasite DNA detection were found in splenic aspirates, followed by skin biopsies and whole blood. CVL-susceptible dogs presented significantly higher frequencies of qPCR positive results than resistant ones employing splenic aspirate as the target of parasite DNA detection (Table 3).

**Table 3:** Frequency of qPCR positive results at any time of the follow-up in visceral leishmaniasis resistant and susceptible dogs.

Sample employed in qPCR for parasite load assessment	Total (n = 148) n (%)	Positive results at least one follow-up time point n (%)		Pearson *X*^2^ test *p-*value
		CVL R (n = 70) n (%)	CVL S (n = 78) n (%)	
Splenic aspirate	100 (67.6)	38 (54.3)	62 (79.5)	0.0011
Skin biopsy	88 (59.5)	42 (60)	46 (59)	0.8990
Whole blood	38 (25.7)	20 (28.6)	18 (23.1)	0.4449

CVL: canine visceral leishmaniasis; R: Resistant; S: Susceptible

Table 4 shows the frequency of qPCR positive results employing spleen aspirates, in CVL-resistant and susceptible dogs during follow-up. qPCR positivity frequencies were calculated at each follow-up time point, considering the animals remaining in the study (lost to follow-up was not considered). CVL-susceptible dogs presented statistically higher frequencies of qPCR positivity than resistant dogs during the study, except in the last time point assessed.

**Table 4:** Frequency of qPCR positive results employing spleen aspirates in visceral leishmaniasis resistant and susceptible dogs, during 24 months of follow-up.

Months after infection diagnosis by qPCR	Positive qPCR % (n/N)		*p*-value*
	CVL R	CVL S	
**0**	54.3% (38/70)	79.5% (62/78)	0.0011
**6**	15.7% (11/70)	41% (32/78)	0.0007
**12**	10.5% (6/57)	41.2% (28/68)	0.0001
**18**	4.7% (2/43)	25% (14/56)	0.0063
**24**	2.5% (1/40)	8.9% (4/45)	0.3644

CVL: canine visceral leishmaniasis; R: Resistant; S: Susceptible

* Fisher’s exact or Pearson *X*^2^

**Fig 2:**
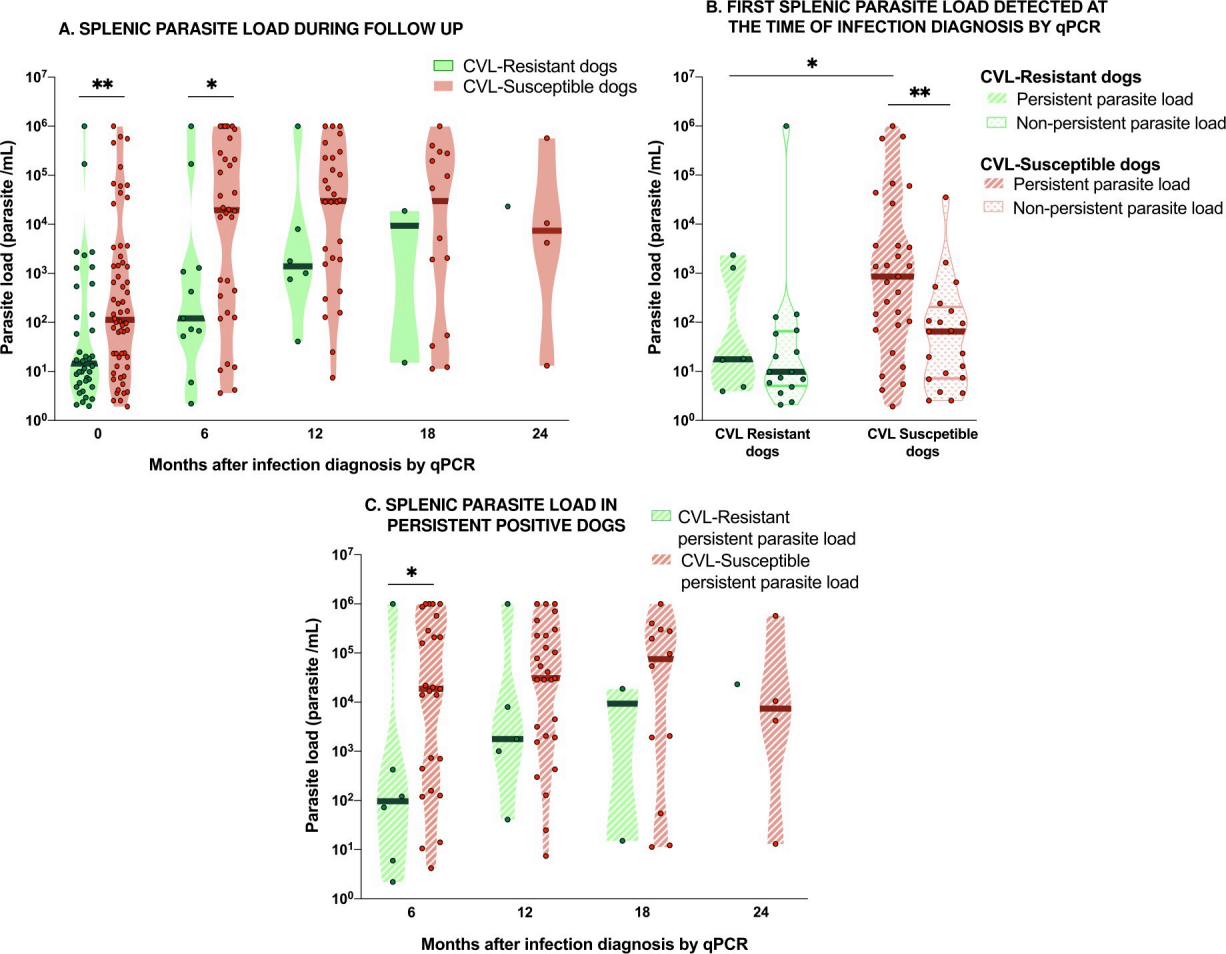
Splenic parasite load during follow-up in canine visceral leishmaniasis (CVL) resistant and susceptible dogs. **(A)** Splenic parasite load of resistant dogs (green dots, light green violin shape) and susceptible dogs (red dots, light red violin shape) assessed at the first time of qPCR infection diagnosis (0) and 6, 12, 18, and 24 months after first qPCR infection diagnosis. **(B)** First splenic parasite load detected by qPCR at the time of infection diagnosis of resistant dogs that would be persistent positive during follow-up (green dots, light green hatched violin shape), resistant non-persistent positive dogs (green dots, clear green hatched violin shape), susceptible dogs that would be persistent positive during follow-up (red dots, light red hatched violin shape), and susceptible dogs that would be non-persistent positive (red dots, clear red hatched violin shape). **(C)** Splenic parasite load of persistent positive susceptible dogs (red dots, light red hatched violin shape) and persistent positive resistant dogs (green dots, light green hatched violin shape) assessed at 6, 12, 18, and 24 months after first qPCR infection diagnosis. Only positive qPCR animals were included in these analyses. Asterisks indicate statistically significant differences (* p<0.05; ** p<0.*01*—Mann Whitney test).

Parasite load varied in all tissues assessed throughout the study period. S2 Fig shows the variations of parasite load in splenic aspirates and skin biopsies of each CVL-resistant or susceptible dogs. Susceptible dogs presented significantly higher parasite load in splenic aspirate than resistant animals at the time of the first diagnosis by qPCR (time point 0) as well as 6 months post-diagnosis (Fig 2A). No significant differences in splenic parasite loads were seen in the other time points. The number of dogs displaying qPCR positive results decreased along with the follow-up, however in CVL-resistant ones, this decrease was more accentuated, and this feature affected the power of the analysis between the groups in the latest time points. No differences were observed in parasite loads between the groups analyzing skin biopsies and whole blood during the study.

We analyzed the persistence of positive qPCR results between CVL-susceptible and resistant dogs. The frequency of susceptible dogs showing persistence of parasites in splenic aspirate was statistically higher (37.2%; 29/78) than resistant dogs (8.6%; 6/70; *p*-value = 0.02; Fisher exact test). In other tissues analyzed, no differences were observed regarding the persistence of parasites between the groups. Fig 2B displays parasite load in splenic aspirate evaluated at the first infection diagnosis by qPCR in persistent and non-persistent susceptible and resistant dogs. Parasite load in persistent susceptible dogs was significantly higher than in non-persistent susceptible ones and in persistent resistant dogs. No significant difference was observed in the parasite load of resistant dogs regarding positivity persistence groups. However, in persistent positive dogs, splenic parasite load of susceptible animals was significantly higher than in resistant ones at 6 months after infection diagnosis by qPCR (Fig 2C). In other time points of the follow-up, no difference was observed between persistent groups.

### Biological mediator assessments in the serum of CVL-resistant and susceptible dogs

Serum levels of several biological mediators were measured in 13 CVL-resistant dogs and 18 susceptible dogs, both 6 to 12 months prior to *L*. *infantum* infection diagnosis (BID—Before Infection Diagnosis), at the time of infection diagnosis (ID), and 6 to 12 months after the first diagnosis (AID—After Infection Diagnosis).

**Fig 3:**
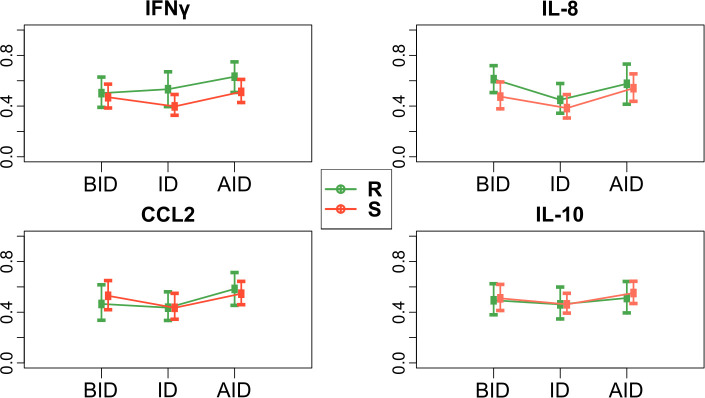
IFNγ, IL-8, CCL2, and IL-10 relative treatment effects (RTE) of CVL-Resistant (R) and susceptible (S) dogs during follow-up. BID: Before Infection diagnosis, ID: Infection Diagnosis, AID: After Infection Diagnosis. RTE are displayed with corresponding 95% confidence intervals.

S3 Fig shows the variations of individual serum levels of biological mediators (pg/mL) of each resistant and susceptible dog during follow-up. No differences were observed for all biological mediators levels comparing resistant and susceptible dogs. To analyze the difference between the groups and during the follow-up, we employed the relative treatment effects (RTE). In this non-parametric longitudinal analysis, an increase in these effects over time indicates the increase in biological mediators’ levels. Fig 3 depicts IFNγ, IL-8, CCL2, and IL-10 RTE in CVL-resistant and susceptible dogs during follow-up. No statistically significant interaction between group and time point was observed, i.e., two groups’ time profiles are parallel. Besides that, no differences were observed between serum levels for all the biological mediators comparing resistant and susceptible groups. However, IFNγ, CCL2 and IL-8 showed statistically significant alterations in serum levels across time (*p*<0.05).

**Fig 4:**
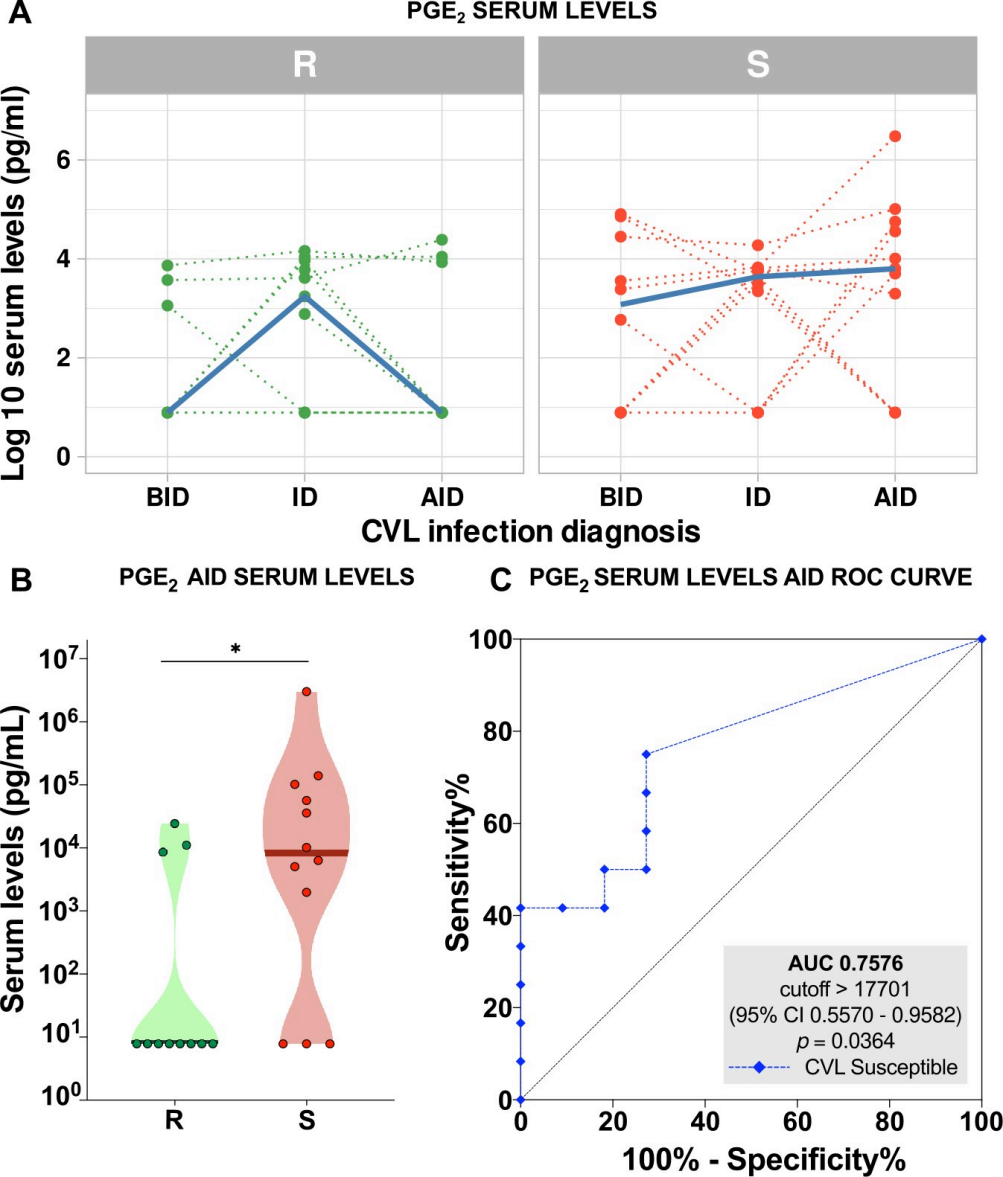
Prostaglandin E_2_ (PGE_2_) as a parameter of disease progression. (**A**) Individual serum levels of PGE_2_, expressed as log10 pg/mL, of canine visceral leishmaniasis (CVL) resistant (R) (green dotted lines) and susceptible (S) (red dotted lines) dogs before infection diagnosis (BID), at infection diagnosis (ID), and after infection diagnosis (AID). Serum levels medians are depicted as thicker solid blue lines. (**B**) PGE_2_ serum levels in CVL-resistant (R) (green dots, n = 11) and susceptible (S) (red dots, n = 12) dogs measured at 6 or 12 months after diagnosis of *Leishmania infantum* infection (AID); asterisk indicates statistically significant differences (*p* = 0.0273—Mann Whitney test U = 32). **(C)** ROC curve analysis to quantitatively estimate the performance of PGE_2_ serum levels AID in identifying CVL-susceptible dogs after infection diagnosis. AUC: area under the curve; CI: confidence interval.

When comparing biological mediators’ serum levels, PGE_2_ presented similar behavior between resistant and susceptible dogs during the follow-up (Fig 4A). However, when considering the time point AID, there was a significant difference between the groups (Fig 4B). Considering this data, PGE_2_ serum levels ROC curve analysis presented 76% accuracy (AUC) in segregating CVL-resistant and susceptible dogs AID (Fig 4C).

### Network analysis of circulating biological mediators in dogs

**Fig 5:**
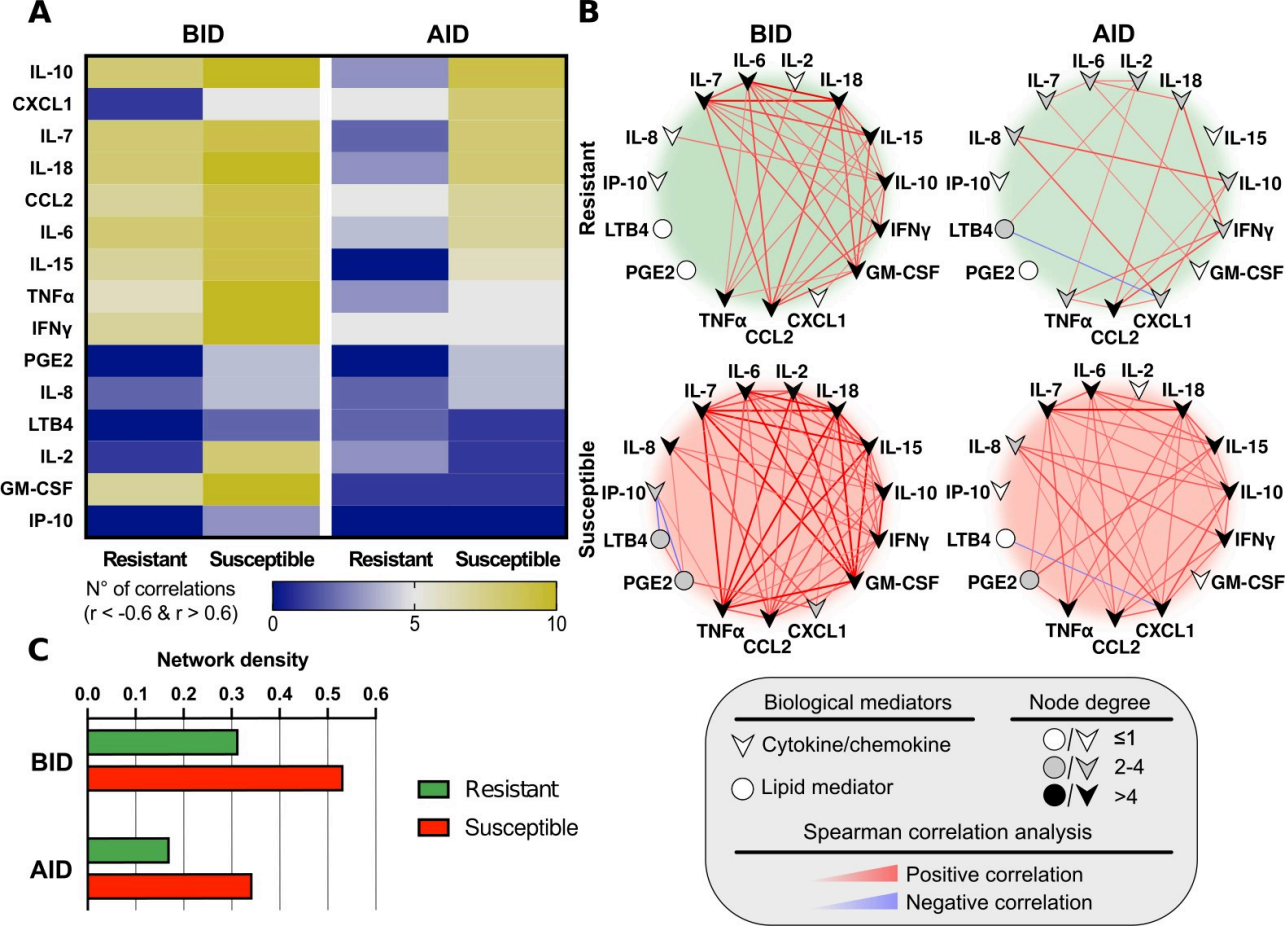
Network analysis of biological mediators in canine sera. (**A**) Heatmap depicting strong statistically significant correlations (Spearman’s test, r ≥ 0.6 or r ≤ -0.6, *p* < 0.05) between the biological mediators measured in CVL-resistant and susceptible canine serum, at 6–12 months prior to detection of *Leishmania infantum* (BID: Before Infection Diagnosis), and 6–12 months after the time of diagnosis (AID: After Infection Diagnosis). (**B**) Network analysis (host interactome) showing interactions between the strong statistically significant correlations (Spearman’s test, r≥0.6, *p*<0.05) between the biological mediators measured in CVL-resistant and susceptible dogs before (BID) and after (AID) infection diagnosis. Positive correlations are shown in red, while blue denotes negative correlations. (**C**) Comparison of Network densities (nodes analysis) of CVL-resistant and susceptible dogs, before and after infection diagnosis.

Interactome analysis shows interactions of biological mediators in each group, represented by the significant number, intensity, and direction of correlations, allowing to observe the immune response dynamics at distinct time points of the study. The relationships in the networks are different between CVL susceptible and resistant dogs, as well as before and after the infection diagnosis in both groups (Fig 5A and 5B). CVL-susceptible dogs presented a higher number of strong correlations, amongst the biological mediators, before and after infection diagnosis compared to resistant dogs (Fig 5A and 5B). In all groups, most of the observed statistically significant correlations were positive (Fig 5B). Interestingly, resistant dogs did not display any participation of PGE_2_ in the networks before and after infection diagnosis, while in susceptible animals, PGE_2_ was involved in the two time-points. We also observed a change in the pattern of correlations concerning PGE_2_ before and after infection diagnosis (Fig 5B). PGE_2_ presented positive correlations with IL-8, CXCL1, and LTB_4_, in susceptible dogs before infection diagnosis and a negative one with IP-10. After infection diagnosis, PGE_2_ showed only positive correlations with pro-inflammatory cytokines such as TNFα, IL-2, IL-15.

We detected an accentuated decrease in the number of correlations of IFNγ in the susceptible dogs after infection diagnosis. Concerning IL-10 correlations, this cytokine presented the same participation before and after the infection diagnosis in the susceptible group. On the other hand, the resistant group displayed a decrease in the involvement of both IFNγ and IL-10 in the network after infection diagnosis.

Moreover, quantifying the number of significant correlations observed in each marker (node analysis in network density) corroborated that the overall number of connections was different between CVL-resistant and susceptible dogs before and after infection diagnosis (Fig 5C). In fact, the number of correlations decreased after infection diagnosis and was more accentuated in the resistant dogs (Fig 5C).

### Dynamics of vector exposure and incidence of *L*. *infantum* infection

**Fig 6:**
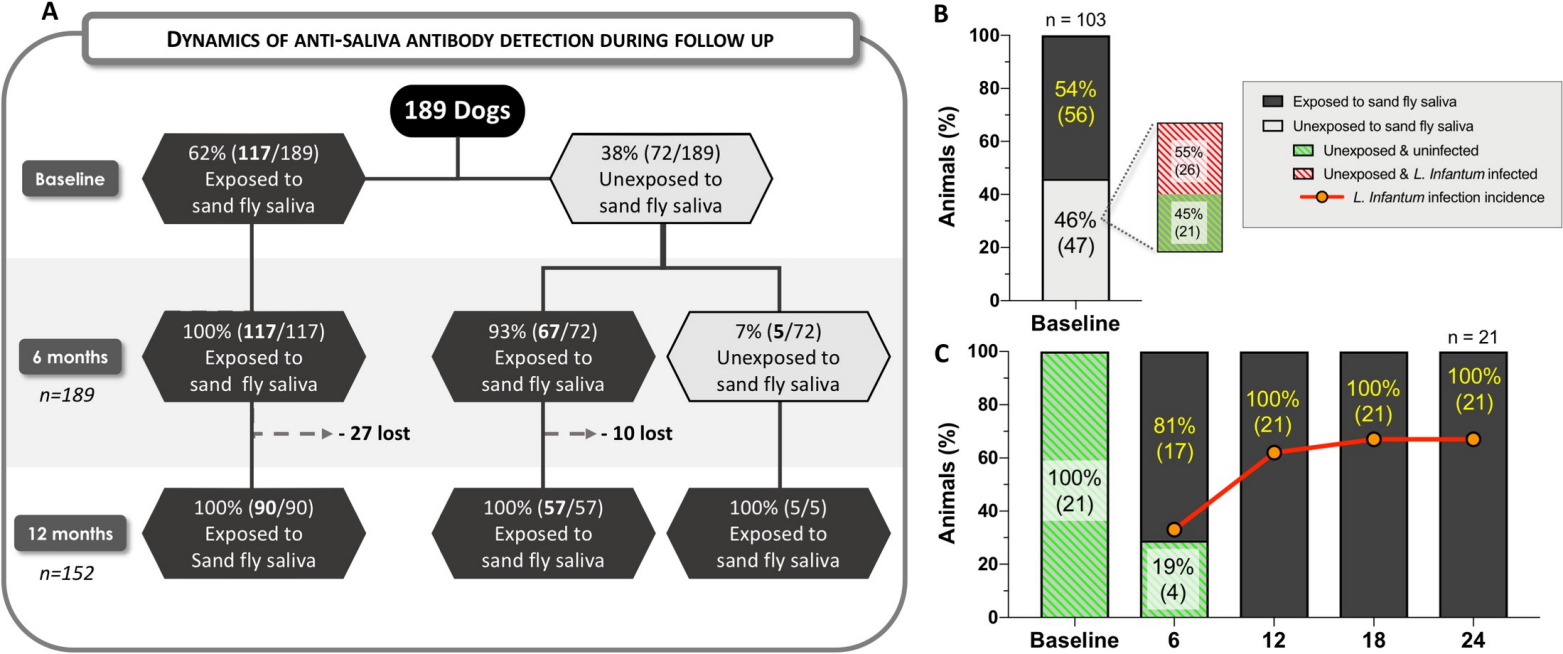
Dynamics of anti-saliva antibody detection and incidence of *Leishmania infantum* infection. A total of 189 dogs were followed for 24 months, with anti-saliva antibodies measured at six-month intervals by rLJM11+17 ELISA. (**A**) Dynamics of anti-saliva antibody detection of all 189 dogs during the two-year follow-up. Light gray hexagons represent the total number of dogs testing negative for anti-saliva antibodies at each follow-up point; Dark grey hexagons represent animals demonstrating positivity for anti-saliva antibodies; Dotted lines represent a loss to follow-up. (**B**) Percentages of the 103 dogs that completed the two-year follow-up period demonstrated positivity or negativity for anti-saliva antibodies at baseline. (**C**) Dynamics of anti-saliva antibody detection and *L*. *infantum* infection incidence in 21 uninfected dogs negative for anti-saliva antibodies at baseline.

We found a strong positive correlation (r = 0.91; *p*<0.0001), and a good agreement (Ƙappa = 0.72; *p*<0.0001) [30] between ELISA results obtained using SGS and those from rLJM11+17. However, when using SGS as the gold standard, rLJM11+17 presented a sensitivity of 81% (95% CI 71–88%) and a specificity of 92% (CI 95% 83–96%) (S4 Fig). Using anti-rLJM11+17 antibody detection as a biomarker of exposure to the vector, we found that 62% (117/189) of the dogs had already been exposed at baseline, while 38% (72/189) were initially determined to be unexposed. Six months after baseline evaluation, 93% (67/72) of the unexposed dogs had become exposed, and by 12 months, all dogs (100%) were considered to have been exposed to the vector (Fig 6A).

Assessing *Lu*. *longipalpis* saliva exposure in the 103 dogs that completed follow-up, 54% (56/103) had already been exposed at baseline, while 46% (47/103) were unexposed to the vector. Among the 47 unexposed dogs, 55% (26/47) had already been infected by *L*. *infantum* at baseline, versus 45% (21/47) non-infected (Fig 6B). A sub-cohort of these 21 dogs negative for both vector exposure and *L*. *infantum* infection at baseline was used to simultaneously evaluate the incidence of vector exposure and *L*. *infantum* infection. An increasing frequency of exposure to sandflies was observed, followed by an increase in *L*. *infantum* infection incidence during follow-up (Fig 6C). In this sub-cohort, after 12 months, 100% (21/21) of the dogs had already been exposed to vector saliva, and 24 months later, 67% (14/21) tested positive for *L*. *infantum* infection.

### Higher sandfly exposure in CVL-susceptible dogs

**Fig 7:**
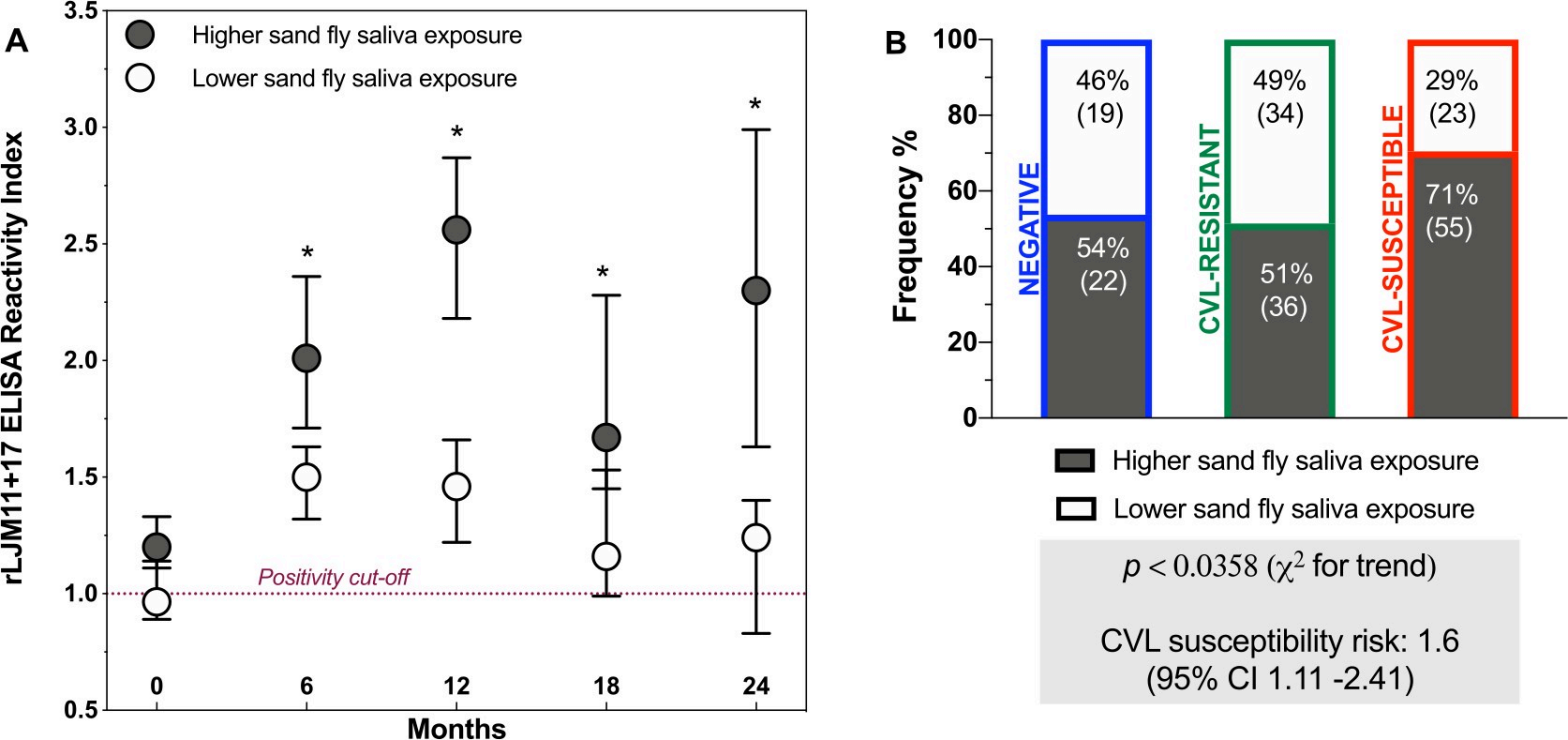
Anti-saliva antibodies levels. (**A**) rLJM11+17 ELISA reactivity index (RI) during the follow-up period; dots represent median RI; bars represent 95% confidence interval (CI) of the median, asterisks indicate significant differences between higher and lower anti-saliva antibody levels (p<0.05, Kruskal-Wallis and Dunn tests). (**B**) Frequencies of negative, CVL-resistant, and susceptible dogs stratified by levels of anti-saliva antibodies; chi-square test for trend (*x*^*2*^) analysis and relative risk (RR) with 95% CI calculated to assess the risk of CVL susceptibility.

Of the 189 animals evaluated at baseline, 60% (113/189) presented higher sandfly exposure at a subsequent time point, while 40% (76/189) did not. Among the animals with higher sandfly exposure, peaks were observed at 6, 12, and 24 months of follow-up compared to baseline (*p*<0.001) (Fig 7A). At all follow-up points, animals classified with higher sandfly exposure presented a higher median rLJM11+17 RI than dogs classified with lower sandfly exposure (*p*<0.05) (Fig 7A). In the group of animals with higher exposure to vectors, 71% were classified as susceptible to CVL, whereas only 51% were considered resistant, indicating a significant difference between these groups (*p*<0,05) (Fig 7B). Moreover, dogs with higher sandfly exposure were 1.6 times (CI: 1.11–2.41) as likely to be classified as susceptible to CVL.

## Discussion

In our cohort study, we are not only comparing animal clinical conditions; dogs were identified as resistant and susceptible regarding CVL disease based on diagnostic results and development of clinical manifestations over time. This classification was performed at the end of the study, and then we attempted to determine associations among CVL susceptibility, parasite load, serum levels and correlations of biological mediators as well as vector exposure intensity during the follow-up.

In longitudinal studies, the loss of follow-up is natural. We had an overall follow-up loss of 45.5%. Dogs from our study lived in an underprivileged area, making them more prone to accidents and death. Therefore, this loss was greater than expected and could have prejudiced the analyses, especially in the last follow-up points.

Reports from the literature have shown correlations between CVL severity and parasite load [31]. In fact, susceptible dogs presented a higher parasite load when first diagnosed and persistence of positive qPCR results during follow-up. It is important to emphasize that parasite load and its persistence were not considered for dogs’ classification in resistant and susceptible groups. Therefore, these findings strengthened our classification.

The immune response is essential for the outcome of the *Leishmania* infection [32]. We assessed biological mediators suggested to influence the infection’s evolution [33], which were also used in our previous study [12] evaluating dogs presenting different degrees of CVL severity. In the present cohort study, we did not find any difference in biological mediators’ serum levels comparing resistant and susceptible dogs during the follow-up. However, in all infected dogs, IFNγ, CCL2 and IL-8 showed significant alterations in serum levels across time. These cytokines have an inflammatory behavior that could reflect a more advanced stage of the infection [34]. Unfortunately, statistical analysis for longitudinal data required larger sample sizes at each time point than in our study. Therefore, with these findings, it was not possible to define a predictive biomarker of resistance or susceptibility to CVL.

Network analysis is a useful approach to visualize the differences in the number of correlations and relationships among biological markers [35,36]. Our correlation analysis allows a more integrated understanding of immune response dynamics in the network format than to the individual analysis of each biological mediator. Interactome analysis and heatmap revealed more interactions in susceptible dogs than in resistant ones, both before and after infection diagnosis. This fact could be associated with the persistence of *L*. *infantum*, and high splenic parasite load, reinforcing data present in the literature concerning different disease stages of CVL [12,37]. In addition, nutritional status, as well as genetic factors, must also be considered in the CVL context [38,39].

The interactome analysis also showed the role played by PGE_2_ in susceptible dogs, demonstrating correlations of this mediator with other ones before and after infection diagnosis, whereas these correlations were not observed in resistant dogs. These findings reinforce the higher levels of serum PGE_2_ concentrations found in susceptible animals after infection diagnosis. Prostaglandin E_2_ is an eicosanoid product of the metabolic pathway of the arachidonic acid cascade [40]. Many reports in the literature have shown the inhibitory function of PGE_2_ on immune responses, contributing to parasite proliferation [40–42]. Accordingly, after infection diagnosis in susceptible dogs, we found higher parasite load and higher serum levels of PGE_2_ compared to resistant dogs.

In our analysis, after infection diagnosis in susceptible dogs, PGE_2_ correlated with TNFα, IL-2, IL-15, and IL-7, most of them with pro-inflammatory functions, possibly attempting to decrease the inflammatory immune response [43–45]. However, this fact also contributes to the increase in the parasite load, reflecting in the disease’s progression, and reinforcing the need for more studies addressing the role of lipid mediators in CVL. In humans, the role played by lipid mediators has already been explored, showing similar results and a possible target for therapeutic approaches [42,46].

IFNγ role in leishmaniasis is well known, increasing macrophages’ ability to destroy parasites [47]. In fact, in susceptible dogs, the interactome analysis showed that IFNγ involvement decreased more sharply after infection diagnosis denoting its importance in controlling the parasite proliferation [47]. In addition, the anti-inflammatory cytokine IL-10 was one of the biological mediators that presented the most correlations in networks under all investigated conditions, except for resistant dogs after infection diagnosis. Since CVL-susceptible animals may fail to control the spread of parasites, IL-10 could contribute to this process. Another hypothesis is that IL-10 involvement may be related to an attempt to control the inflammatory response, albeit unsuccessfully.

The differences observed in the interactome analysis of CVL-resistant and susceptible dogs could also be attributed to co-infections, such as ehrlichiosis and babesiosis. However, these co-infections were highly present in susceptible, resistant, and non-infected dogs. Therefore, these coinfections are possibly not responsible for the differences seen in immune response dynamics between groups. Nevertheless, we cannot rule out the co-infections’ influence on the outcome of CVL [48] since our study population was highly coinfected, and other pathogens were not investigated.

At baseline, 36% of the studied dogs did not present anti-rLJM11+17 antibodies. The rLJM11+17 salivary proteins used in ELISA detection were shown to be highly specific, but with a sensitivity of 81%. Therefore, this sensitivity level could be one of the study limitations since 20% of the animals would not be detected at each time point. However, the longitudinal evaluation could have reduced the impact of this limitation. Importantly, IgG anti-saliva antibodies’ levels were shown to decrease after nine weeks in experimentally exposed dogs [49,50]. Some of the negative results for anti-saliva antibodies in our cohort could reflect decreases in these levels due to elapsed time since exposure. Moreover, 17% of the rLJM11+17-negative dogs were puppies, and 34% of the animals’ owners applied repellent, which could have reduced exposure to sandflies.

Forty-seven dogs that did not present anti-rLJM11+17 antibodies were followed for 24 months. A high percentage of these animals (55%) were already infected at baseline. In addition to the possible explanations described above, we must also consider the vertical transmission of the parasite [51], which we speculate may commonly occur in endemic areas. The other 21 dogs followed for 24 months were rLJM11+17 and *L*. *infantum* infection negative at baseline. In this particular case, the sample size was small since encountering these animals in endemic areas is rare because they are exposed to sandflies all the time. Interestingly, these animals exhibited a higher frequency of exposure to sandflies over a short period in association with an increase in the incidence of *L*. *infantum* infection. Consonantly, Quinnel *et al*. also reported rapid exposure in previously unexposed sentinel dogs when introduced into an endemic area in Brazil [14].

In our study, a large proportion of susceptible dogs presented higher levels of anti-sandfly saliva antibodies, indicating an intense exposure to the vector and suggesting an association between exposure and susceptibility to CVL. This fact could be associated with continuous re-exposure to sandfly bites. However, we cannot rule out that susceptible dogs might present an overactive B cell response and, therefore, increased antibody production, including those against sandfly saliva, independently of vector exposure [52]. Our albumin and globulin ratio analysis showed that susceptible dogs presented a lower ratio, and increased serum globulin. Despite this limitation, there are pieces of evidence in the literature showing a positive association between *P*. *perniciosus* anti-saliva antibody production and active *L*. *infantum* infection [13]. In addition, a significant positive correlation was found between the production of anti-*Leishmania* and anti-*Lu*. *longipalpis* saliva antibodies [14].

One hypothesis is that more exposure to the vector may increase the chance of becoming re-infected, which would lead to susceptibility. However, this hypothesis is difficult to address in a field study since increased parasite load, or anti-*Leishmania* antibody levels could occur even in the absence of reinfection, as *Leishmania* parasites undergo constant replication [53,54]. A second hypothesis is that the mechanism that triggers susceptibility may be related to the silencing of several genes involved in an effective immune response. In fact, a previous study demonstrated that several IFN-inducible genes were up-regulated in response to *Lu*. *intermedia* salivary gland proteins before infection, and we submit that these genes could become activated through multiple sandfly exposure events and then inhibited by *L*. *braziliensis* infection, resulting in uncontrolled infection and more severe disease presentation [38]. However, during a blood meal, the sandfly does not only inoculate *Leishmania* and saliva; other components are also regurgitated [55–57]. These components have already been implicated with the establishment of infection, as well as the severity of the disease. Direct association between sandflies’ gut microbiota and parasite development in the midgut of the vector has been demonstrated [58], and the egestion of these microorganisms together with the parasite may contribute to the severity of leishmaniasis [55]. Studying cutaneous leishmaniasis, Atayde *et al*. demonstrated that *Leishmania* exosomes enhance lesion development by favoring parasite replication [56]. Proteophosophoglycans from *Leishmania* have been proven to increase parasite persistence at the site of the bite [57].

Our results demonstrated that anti-sandfly saliva antibodies could represent an important epidemiological tool to assess vector exposure in endemic areas. Since recombinant proteins present in the salivary extract of *Lu*. *longipalpis* are laborious to obtain, sandfly salivary recombinant proteins such as rLJM11+17 have proven to be a suitable substitute to assess vector exposure. Salivary proteins may prove useful for screening canine sera in areas endemic for VL, thus enhancing the effectiveness of vector control measures, such as the use of repellent-impregnated collars, and also providing better estimations of the risk of *L*. *infantum* transmission [50]. Moreover, using these recombinant proteins could also facilitate recognizing new areas whose presence of the vector has not yet been identified, thus monitoring the possible expansion of VL transmission.

Our data can be helpful to advance understanding of the pathogenesis of VL since dogs are a good model of human disease [59]. Elucidating the VL progress can be important for improving prophylactic and therapeutic strategies. CVL complexity is multifactorial and implies several correlated biological factors. In this study, we evaluated different parameters described in the literature as essential for the disease outcome [60]. This experimental approach is complicated and time-consuming; however, the cohort allowed us to understand the natural infection progression, monitoring parasite load, vector exposure intensity, and biological mediator interactions during follow-up. The animals’ biological responses to the parasite infection varied widely over time, depicting a more natural disease’s general picture, but the data analysis was more complex.

We identified a discriminatory biosignature between the two groups assessing splenic parasite load, interaction of biological mediators, PGE_2_ serum levels, and intensity of exposure to sandfly. All these parameters were elevated in susceptible dogs compared to resistant ones. Our findings reinforce the idea that CVL is a complex multifactorial disease that is affected by a set of factors which are correlated and, for a better understanding of CVL, should not be evaluated in an isolated way.

## Supporting information

S1 Fig Clinical score profile during follow-up of canine visceral leishmaniasis (CVL) resistant (R) and susceptible (S) dogs. Green dotted lines represent each resistant dog clinical score, while red dotted lines represent each susceptible dog during follow-up. Clinical score means are represented by large dotted blue line while clinical score medians are depicted as thicker solid blue line.
(TIF)

S2 Fig Individual parasite loads in spleen aspirate and skin biopsies of canine visceral leishmaniasis (CVL) resistant (R) and susceptible (S) dogs during follow-up. Parasite load was assessed in canine tissues during follow-up by qPCR; (**A**) Parasite load in splenic aspirate, expressed as parasites/mL; (**B**) Parasite load in skin biopsies, expressed as parasites/100 mg of tissue. Green dotted lines represent each resistant dog parasite load during follow-up, while red dotted line each susceptible dog. Parasite load means are represented by large dotted blue lines while parasite load medians are depicted as thicker solid blue lines.
(TIF)

S3 Fig Individual serum levels of biological mediators in canine visceral leishmaniasis (CVL) Resistant (R) and Susceptible (S) dogs during follow-up. Serum levels of biological mediators were expressed as log10 pg/mL and measured before infection diagnosis (BID), at infection diagnosis (ID), and after infection diagnosis (AID). CVL-resistant (R) dogs are depicted as green dotted lines (n = 11) and susceptible (S) ones as red dotted lines (n = 12). Serum levels medians are depicted as thicker solid blue lines.
(TIF)

S4 Fig Validation of the recombinant proteins LJM11+17 as biomarkers of sand fly exposure. A total of 157 sera obtained from animals in an endemic area were tested for both anti-SGS and anti-rLJM11+17 antigens in separate ELISA assays. Reactivity Index (RI) values corresponding to antibody detection were compared. (**A**) Correlation between anti-SGS and anti-rLJM11+17 RIs detected by ELISA; Relevant Spearman correlation coefficients (r) and *p*-values are shown. (**B**) Diagnostic performance parameters represented as percentages with 95% confidence intervals (CI) of rLJM11+17 ELISA compared to ELISA using SGS antigens; PPV: positive predictive value; NPV: negative predictive value: Kappa: Kappa agreement index; *x*^*2*^: Pearson chi-square test.
(TIFF)

S1 Table Comparison of serum albumin and globulin concentrations between resistant and susceptible *Leishmania infantum* infected dogs at 6, 12, 18, and 24 months after infection diagnosis. (DOCX)
