# Supplementary figures and images for: Immune response dynamics and Lutzomyia longipalpis exposure characterize a biosignature of visceral leishmaniasis susceptibility in a canine cohort
Source: PLoS Negl Trop Dis. 2021 Feb 22;15(2):e0009137. doi: 10.1371/journal.pntd.0009137 (PMC7943000; doi:10.1371/journal.pntd.0009137)

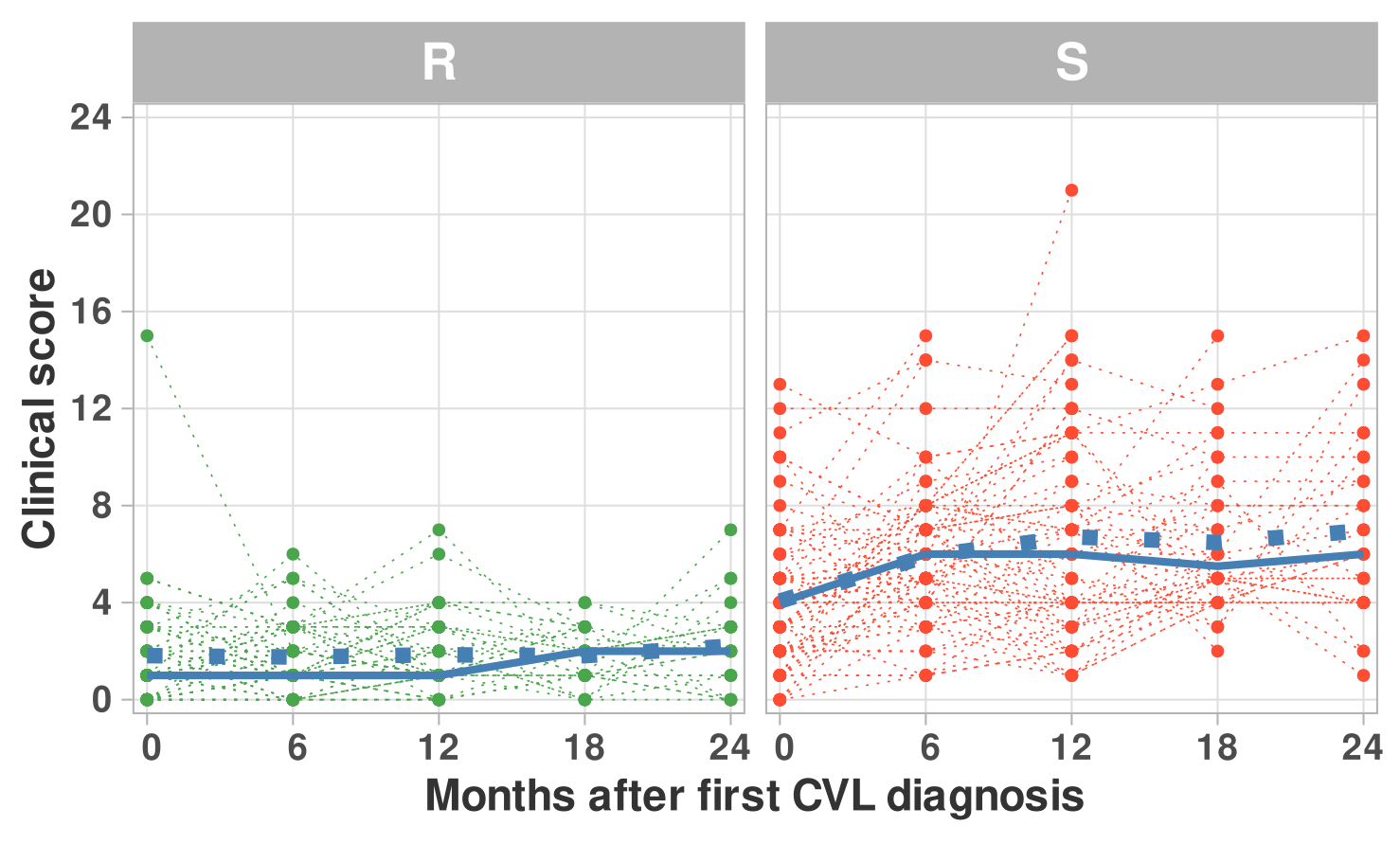

Supplement: S1 Fig — Green dotted lines represent each resistant dog clinical score, while red dotted lines represent each susceptible dog during follow-up. Clinical score means are represented by large dotted blue line while clinical score medians are depicted as thicker solid blue line. (TIF) [file pntd.0009137.s001.tif]

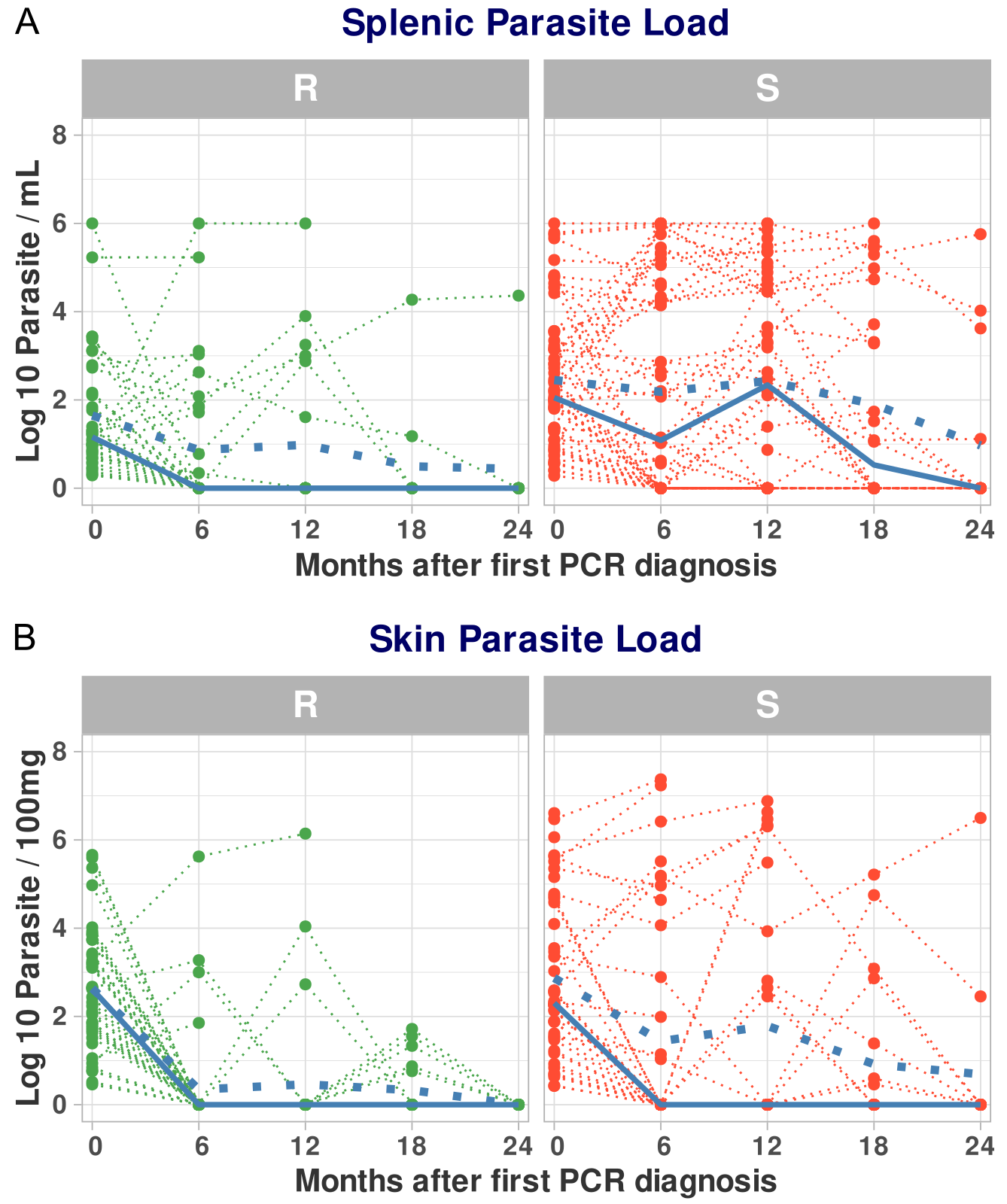

Supplement: S2 Fig — Parasite load was assessed in canine tissues during follow-up by qPCR; (A) Parasite load in splenic aspirate, expressed as parasites/mL; (B) Parasite load in skin biopsies, expressed as parasites/100 mg of tissue. Green dotted lines represent each resistant dog parasite load during follow-up, while red dotted line each susceptible dog. Parasite load means are represented by large dotted blue lines while parasite load medians are depicted as thicker solid blue lines. (TIF) [file pntd.0009137.s002.tif]

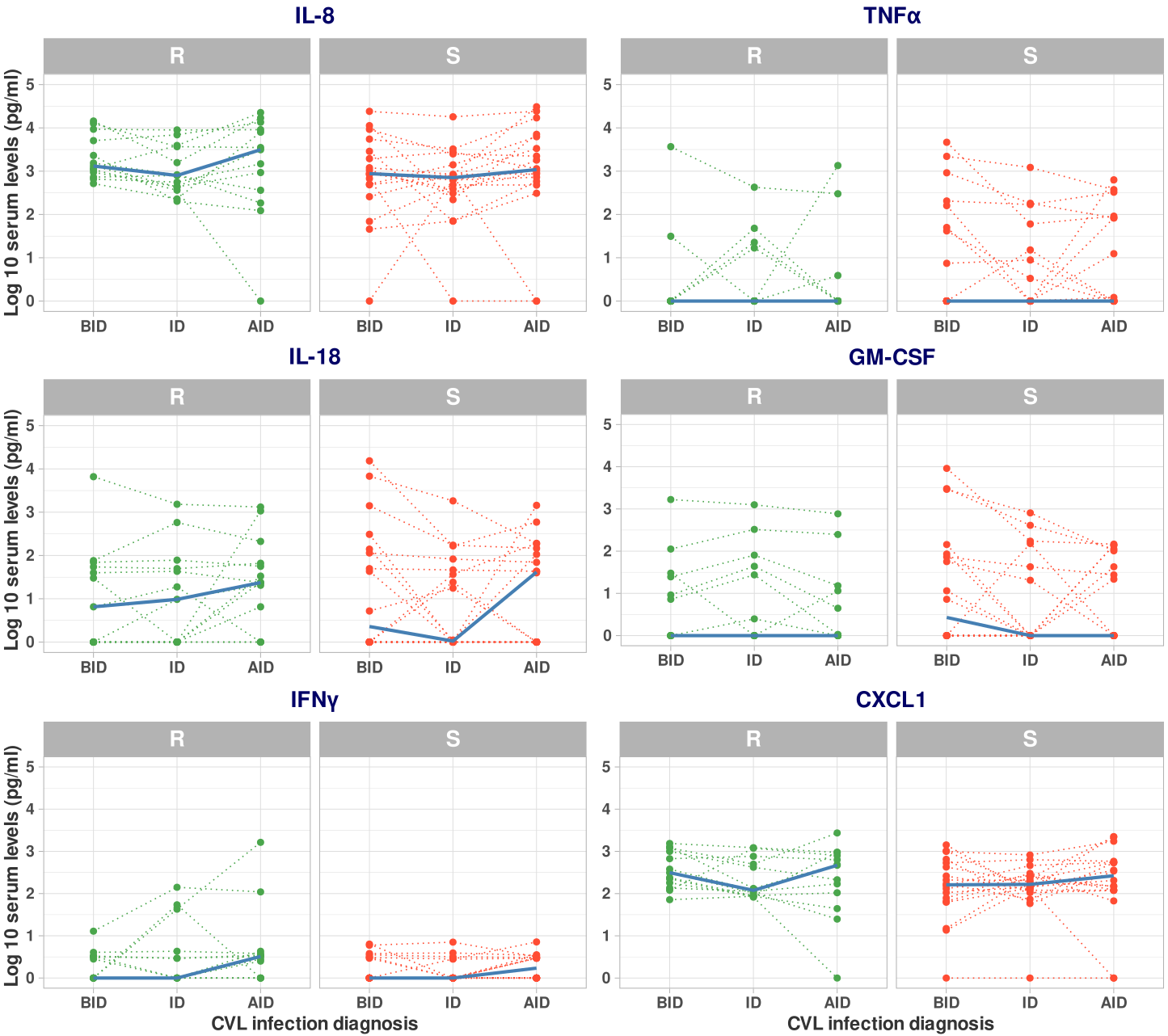

Supplement: S3 Fig — Serum levels of biological mediators were expressed as log10 pg/mL and measured before infection diagnosis (BID), at infection diagnosis (ID), and after infection diagnosis (AID). CVL-resistant (R) dogs are depicted as green dotted lines (n = 11) and susceptible (S) ones as red dotted lines (n = 12). Serum levels medians are depicted as thicker solid blue lines. (TIF) [file pntd.0009137.s003.tif]

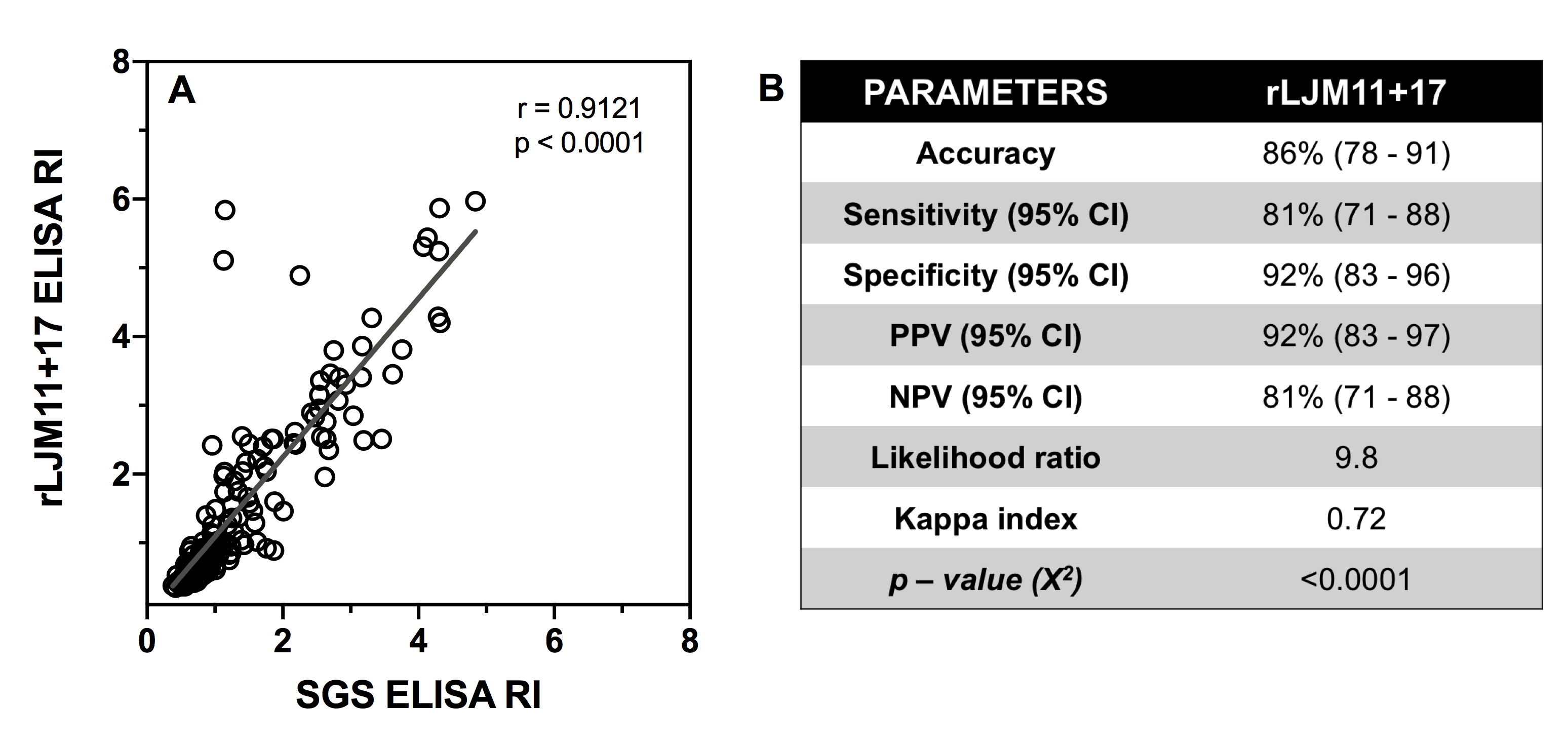

Supplement: S4 Fig — A total of 157 sera obtained from animals in an endemic area were tested for both anti-SGS and anti-rLJM11+17 antigens in separate ELISA assays. Reactivity Index (RI) values corresponding to antibody detection were compared. (A) Correlation between anti-SGS and anti-rLJM11+17 RIs detected by ELISA; Relevant Spearman correlation coefficients (r) and p-values are shown. (B) Diagnostic performance parameters represented as percentages with 95% confidence intervals (CI) of rLJM11+17 ELISA compared to ELISA using SGS antigens; PPV: positive predictive value; NPV: negative predictive value: Kappa: Kappa agreement index; x2: Pearson chi-square test. (TIFF) [file pntd.0009137.s004.tiff]
