# Supplementary material for: Immune response dynamics and Lutzomyia longipalpis exposure characterize a biosignature of visceral leishmaniasis susceptibility in a canine cohort
Source: PLoS Negl Trop Dis. 2021 Feb 22;15(2):e0009137. doi: 10.1371/journal.pntd.0009137 (PMC7943000; doi:10.1371/journal.pntd.0009137)
Supplement: S1 Table — (DOCX) [file pntd.0009137.s005.docx]

**S1 Table.** **Comparison of serum albumin and globulin concentrations between resistant and susceptible *Leishmania infantum* infected dogs at 6, 12, 18, and 24 months after infection diagnosis**

| **Months after infection diagnosis** | **Albumin (mg/dl)**  **median ± IQR** | | |  | **Globulin (mg/dl)**  **median ± IQR** | | |  | **Albumin and globulin ratio**  **median ± IQR** | | |
| --- | --- | --- | --- | --- | --- | --- | --- | --- | --- | --- | --- |
|  | **R** | **S** | ***p* value** |  | **R** | **S** | ***p* value** |  | **R** | **S** | ***p* value** |
| **6** | 2.8 ± 0.83 | 2.38 ± 0.64 | ** |  | 5.33 ± 2.8 | 5.86 ± 2.29 | ns |  | 0.49 ± 0.31 | 0.39 ± 0.19 | * |
| **12** | 2.71 ± 0.88 | 2.3 ± 0.84 | *** |  | 5.39 ± 1.81 | 6.66 ± 2.58 | ** |  | 0.51 ± 0.31 | 0.35 ± 0.19 | *** |
| **18** | 2.90 ± 0.51 | 2.55 ± 0.77 | * |  | 5.11 ± 2.51 | 6.31 ± 1.98 | ** |  | 0.54 ± 0.27 | 0.40 ± 0.23 | ** |
| **24** | 2.99 ± 0.66 | 2.57 ± 0.65 | ns |  | 4.98 ± 2.61 | 6.16 ±2.39 | ns |  | 0.57 ± 0.42 | 0.41 ± 0.22 | * |

IQR: Interquartile range; R: Resistant; S: Susceptible

*p*-value - Mann Whitney test: (*) p < 0.05; (**) p < 0.01; (***) p < 0.001; ns – not significant
